# A Prospective Study on Fixation of Syndesmotic Ankle Injury: Tight Rope Versus Screw Fixation

**DOI:** 10.7759/cureus.67172

**Published:** 2024-08-19

**Authors:** Ishan Shevate, Rahul Salunkhe, Faiz Pervez, Prashant Pawar

**Affiliations:** 1 Orthopaedics, Dr. D. Y. Patil Medical College, Hospital and Research Center, Pune, IND

**Keywords:** treatment outcome, orthopedic procedures, internal, fracture fixation, syndesmosis, ankle injuries

## Abstract

Background

Syndesmotic injury can result in significant instability and long-term complications if not treated correctly. Traditional management has involved transyndesmotic screw fixation, but a newer technique, the tight rope system, has been developed to mitigate some of the issues related to screw fixation, such as hardware discomfort and the necessity for hardware removal.

Methods

In this randomized, prospective study, 32 patients with ankle injuries requiring syndesmotic fixation were equally divided into two groups: one receiving the tight rope system (n=16) and the other undergoing screw fixation (n=16). The patients were monitored for six months following surgery. The study measured outcomes such as time to weight-bearing, range of motion, pain levels, functional outcomes using the American Orthopaedic Foot & Ankle Society (AOFAS) Ankle-Hindfoot Scale, and complication rates.

Results

Both groups had comparable demographic and injury profiles. The tight rope group achieved weight-bearing significantly earlier (6.19 ± 0.9 weeks vs. 7.13 ± 0.95 weeks, p=0.008) and had better functional outcomes at six months (87.5% excellent AOFAS scores vs. 37.5%, p=0.003) compared to the screw fixation group. The range of motion and pain scores were similar between the groups. Different complications were observed: screw breakage was more common in the screw fixation group, while the tight rope group experienced more laxity. Overall complication rates were similar.

Conclusion

Both techniques were effective in reducing pain and maintaining range of motion. However, the tight rope system allowed for earlier weight-bearing and better functional outcomes at six months. These results indicate that the tight rope system may provide certain advantages in treating syndesmotic injuries, although the choice of technique should be tailored to the specific injury and patient factors.

## Introduction

One of the key structures that stabilize the ankle is the syndesmosis. External rotation can cause syndesmotic injuries, which are present in approximately 11-13% of ankle fractures [1]. Syndesmotic ligament injuries, though less common than other types of ankle sprains, can be particularly debilitating and lead to long-term complications if not properly diagnosed and managed. The syndesmosis is a fibrous joint that connects the distal tibia and fibula, comprising four ligaments: the anterior inferior tibiofibular ligament, posterior inferior tibiofibular ligament, transverse tibiofibular ligament, and interosseous ligament [2]. These ligaments work in unison to provide stability and allow a limited degree of movement between the tibia and fibula during ankle dorsiflexion and plantarflexion [3,4].

Syndesmotic injuries generally result from high-energy trauma, such as rotational forces applied to the ankle or hyperdorsiflexion, leading to disruption or tearing of one or more syndesmotic ligaments [5]. Such disruptions can cause instability and misalignment of the ankle mortise, potentially resulting in long-term issues like arthritis, chronic pain, and impaired function if left untreated [6].

Traditionally, the standard treatment for syndesmotic injuries has been open reduction and internal fixation (ORIF) with transyndesmotic screw fixation. This surgical technique aims to restore the anatomical alignment between the tibia and fibula, allowing the ligaments to heal correctly. However, despite its effectiveness in achieving reduction and stabilization, this method can lead to complications such as the need for hardware removal, malreduction, and the development of post-traumatic arthritis [7].

Recently, an alternative technique known as the "tight rope" or suture-button system has gained popularity for treating syndesmotic injuries. This method involves using a flexible suture-button that is passed through drill holes in the fibula and tibia, providing dynamic fixation while allowing physiological movement of the syndesmosis during ankle motion [8]. This technique is favored by some experts because it may reduce complications associated with transyndesmotic screws, enable earlier weight-bearing and rehabilitation, and potentially improve functional outcomes.

Despite the growing use of the tight rope system, there is a lack of high-quality, prospective studies directly comparing its efficacy and outcomes with the traditional transyndesmotic screw fixation method. This prospective study aims to fill this gap by thoroughly evaluating and comparing the clinical and radiological outcomes, as well as patient-reported functional scores, between the two fixation techniques in treating syndesmotic injuries. By providing valuable comparative analysis, this research may help guide surgical decision-making and optimize patient care for this challenging injury.

With this context, our study aims to compare the outcomes of syndesmotic fixation in ankle injuries using the tight rope system versus syndesmotic screw fixation and to evaluate the functional outcomes of these ankle injuries.

## Materials and methods

Study design and setting: This randomized prospective study was conducted at the Department of Orthopedics in a tertiary hospital. The research was conducted over 18 months, from August 2022 to February 2024, following ethical approval from the Institutional Ethics Committee.

Patient selection and randomization: The study included 32 patients with ankle injuries, this sample size was determined using WinPepi software (www.brixtonhealth.com) based on a prevalence of 70%, a 95% confidence interval, and a 16% acceptable error. Participants were adults aged 18-70 years with Danis-Weber type B and C closed ankle fractures. Exclusion criteria included osteoporosis, other lower limb fractures, ankle arthritis, and pre-existing abnormal ankle function. After obtaining written informed consent, patients were randomly assigned to two groups of 16 using a lottery method: Group A (Screw Fixation) and Group B (Tight Rope fixation).

Surgical procedures: Surgeries were performed under spinal anaesthesia with patients in a supine position on X-ray translucent tables, using a padded thigh tourniquet and following the AO technique. For Group A, a longitudinal lateral incision along the fibula was made, with superficial dissection between the peroneus tertius anteriorly and the peroneus longus and brevis posteriorly. Deep dissection involved periosteum release at the plate application site. One-third of tubular plates were used for fibular fracture fixation. In Group B, the tight rope system was implemented using a needle-guided technique to pass the device through drill holes from the lateral to the medial side of the ankle. Fluoroscopy was used to confirm proper positioning and reduction.

Post-operative care and rehabilitation: Post-operative care included antibiotics and analgesics for all patients. Group A patients were non-weight-bearing with a below-knee back splint for six weeks, followed by a range of motion exercises for another six weeks. Syndesmotic screws were typically removed at 12-16 weeks, after which full weight-bearing was initiated. Group B patients were non-weight-bearing with a below-knee back splint for three weeks, followed by three weeks of range of motion exercises, and then gradually progressed to full weight-bearing.

Outcome measures: Primary outcome measures included the modified American Orthopaedic Foot & Ankle Society (AOFAS) and the Karlsson scoring system. Pain was assessed using a Verbal Analog Scale. The accuracy of reduction was evaluated radiologically as good, fair, or poor. Functional outcomes were measured at baseline and during follow-up visits at six weeks, three months, six months, and 12 months post-surgery. Complications related to the surgical procedures were carefully monitored and documented throughout the study period.

Data analysis: Data was entered into Excel and analyzed using SPSS version 20.0 (IBM Corp., Armonk, New York). Quantitative data were reported as mean, median, standard deviation, and ranges, while qualitative data were expressed as frequencies and percentages. The Student's t-test (two-tailed) was used to compare means between the two groups, with a p-value <0.05 considered statistically significant.

## Results

Demographic and clinical characteristics were similar between the two groups (Table 1), with no statistically significant differences in age distribution, gender, body mass index (BMI), laterality of injury, or mode of injury. The following statistical analysis for all the tables is done using the Chi-square test/Fisher's exact test.

**Table 1:** Demographic and Clinical Characteristics BMI: Body Mass Index, RTA: Road Traffic Accidents

Characteristic	Tight Rope (n=16)	Screw Fixation (n=16)	p-value
Age (most common)	20-30 & 51-60 years (31.2% each)	20-30 years (31.2%)	0.88
Gender (male)	62.5%	50%	0.47
BMI (overweight)	50%	43.8%	0.96
Right-sided injury	68.8%	62.5%	0.71
RTA as mode of injury	75%	56.2%	0.32

Surgical duration was slightly longer in the Tight Rope group, but the difference was not statistically significant (p=0.58). The post-operative range of motion (dorsiflexion and plantar flexion) was comparable between the two groups, with no significant differences. Pain scores (Visual Analog Scale, VAS) were similar in both groups post-operatively. Time to weight bearing was significantly shorter in the Tight Rope group (6.19 weeks) compared to the Screw Fixation group (7.13 weeks), p=0.008. Functional outcomes at six months, as measured by the AOFAS score, were significantly better in the Tight Rope group, with 87.5% achieving excellent outcomes compared to 37.5% in the Screw Fixation group (p=0.003)(Table 2).

**Table 2:** Surgical and functional outcomes Data are presented as mean ± SD (mean ± 95% CI) for various outcomes in the Tight Rope (n=16) and Screw Fixation (n=16) groups. (*) denotes p-values with significant differences between the groups, where p-values are less than 0.05. VAS: Visual Analog Scale; AOFAS: American Orthopaedic Foot & Ankle Society.

Outcome Measure	Tight Rope (n=16)	Screw Fixation (n=16)	p-value
Duration of Surgery	91.56 ± 22.4 min (80.6, 102.5)	86.8 ± 24.8 min (77.08, 96.52)	0.58
Post-op Dorsiflexion	17.6 ± 1.5° (16.87, 18.33)	17.3 ± 1.6° (16.32, 18.28)	0.57
Post-op Plantar Flexion	39.5 ± 1.6° (38.52, 40.48)	39.4 ± 1.5° (38.67, 40.13)	0.91
Post-op VAS	1.9 ± 0.7 (1.63, 2.17)	2.0 ± 0.81 (1.75, 2.25)	0.82
Time to Weight Bearing	6.19 ± 0.9 weeks (5.91, 6.47)	7.13 ± 0.95 weeks (6.84, 7.42)	0.008*
Excellent AOFAS Score at six Months	87.5% (CI not provided)	37.5% (CI not provided)	0.003*

Complications differed between the groups, with screw breakage (25%) being the most common in the Screw Fixation group, while laxity in tight ropes (25%) was the primary complication in the Tight Rope group. However, the overall complication rates were not statistically significantly different between the groups (Table 3).

**Table 3:** Complications in both the groups

Complication	Tight Rope (n=16)	Screw Fixation (n=16)
Wound dehiscence	6.2%	6.2%
Superficial surgical site infection	6.2%	12.5%
Screw breakage	0%	25%
Laxity in the tight ropes	25%	0%

Our results suggest that while both techniques are effective, the Tight Rope system may offer advantages in terms of earlier weight bearing and better functional outcomes at six months post-surgery.

## Discussion

Ankle fractures are prevalent injuries managed by orthopedic surgeons, with an incidence rate of 187 per 100,000 individuals. This rate is expected to rise due to an aging population, increasing obesity rates, and higher levels of athletic participation [9]. Syndesmotic injuries, or high ankle sprains, present significant challenges and necessitate appropriate management for optimal recovery. This study compared two surgical approaches for treating these injuries: the tight rope system and transyndesmotic screw fixation.

Traditional screw fixation has long been the standard treatment but comes with challenges such as hardware discomfort, stiffness, and the risk of screw breakage. The tight rope system, which utilizes a suture endobutton design, aims to mitigate these issues [8]. Our findings indicated no significant differences in demographic characteristics or injury patterns between the two groups, ensuring a reliable comparison of surgical outcomes. Operative times were similar across both groups, consistent with previous research [10,11].

Postoperative range of motion and pain levels, as measured by the Visual Analog Scale (VAS), were comparable for both techniques, in line with the results reported by Cottom et al. [12]. However, the Tight Rope group exhibited a significantly shorter time to weight-bearing, corroborated by several studies indicating faster functional recovery with this system [13,14]. Functional outcomes, evaluated using the AOFAS scoring system at the six-month mark, were notably better in the Tight Rope group. This finding aligns with previous research and may be attributed to the dynamic stabilization provided by the tight rope system.

While complication rates were similar between the groups, the nature of the complications differed. Screw breakage was more common in the screw fixation group, whereas laxity was the predominant issue in the Tight Rope group. These results are consistent with existing literature [8,15-17]. Bawady et al. reported similar complication rates between techniques, with specific issues such as malreduction, late diastasis, and ankle joint stiffness occurring in both groups [13].

Limitations

This study has a few limitations. It involves a small sample size of 32 patients and a short follow-up period of only six months, which may not capture long-term outcomes. Conducted at a single medical center, its findings may not be widely applicable. The exclusion of patients with certain comorbidities further limits generalizability. Additionally, the lack of blinding and potential bias in the randomization method could affect the results. Finally, the study does not thoroughly discuss the severity and impact of complications or account for the variability in surgical technique.

## Conclusions

This study evaluates the efficacy of the tight rope system versus transyndesmotic screw fixation for treating syndesmotic injuries. In conclusion, while both methods are effective, the tight rope system has notable advantages, including faster weight-bearing and better functional outcomes at six months. Although complication rates are similar for both techniques, screw fixation is more prone to issues like screw breakage, whereas the tight rope system mainly presents with laxity. Due to its dynamic stabilization benefits, the tight rope system is a promising alternative. Treatment choice should be guided by the specifics of the injury, the surgeon's expertise, and patient preferences. Further research with larger sample sizes and extended follow-up periods is necessary to confirm these results and address the study’s limitations.

## References

[1] Thur CK, Edgren G, Jansson KÅ, Wretenberg P (2012). Epidemiology of adult ankle fractures in Sweden between 1987 and 2004: a population-based study of 91,410 Swedish inpatients. Acta Orthop.

[2] Hermans JJ, Beumer A, de Jong TA, Kleinrensink GJ (2010). Anatomy of the distal tibiofibular syndesmosis in adults: a pictorial essay with a multimodality approach. J Anat.

[3] Ogilvie-Harris DJ, Reed SC, Hedman TP (1994). Disruption of the ankle syndesmosis: biomechanical study of the ligamentous restraints. Arthroscopy.

[4] Xenos JS, Hopkinson WJ, Mulligan ME, Olson EJ, Popovic NA (1995). The tibiofibular syndesmosis. Evaluation of the ligamentous structures, methods of fixation, and radiographic assessment. J Bone Joint Surg Am.

[5] Nielson JH, Gardner MJ, Peterson MG, Sallis JG, Potter HG, Helfet DL, Lorich DG (2005). Radiographic measurements do not predict syndesmotic injury in ankle fractures: an MRI study. Clin Orthop Relat Res.

[6] van den Bekerom MP, Lamme B, Hogervorst M, Bolhuis HW (2007). Which ankle fractures require syndesmotic stabilization?. J Foot Ankle Surg.

[7] Kaye RA (1989). Stabilization of ankle syndesmosis injuries with a syndesmosis screw. Foot Ankle.

[8] Naqvi GA, Shafqat A, Awan N (2012). Tightrope fixation of ankle syndesmosis injuries: clinical outcome, complications and technique modification. Injury.

[9] Johansson H, Kanis JA, Odén A (2014). A meta-analysis of the association of fracture risk and body mass index in women. J Bone Miner Res.

[10] Kortekangas T, Savola O, Flinkkilä T (2015). A prospective randomised study comparing TightRope and syndesmotic screw fixation for accuracy and maintenance of syndesmotic reduction assessed with bilateral computed tomography. Injury.

[11] Laflamme M, Belzile EL, Bédard L, van den Bekerom MP, Glazebrook M, Pelet S (2015). A prospective randomized multicenter trial comparing clinical outcomes of patients treated surgically with a static or dynamic implant for acute ankle syndesmosis rupture. J Orthop Trauma.

[12] Cottom JM, Hyer CF, Philbin TM, Berlet GC (2009). Transosseous fixation of the distal tibiofibular syndesmosis: comparison of an interosseous suture and endobutton to traditional screw fixation in 50 cases. J Foot Ankle Surg.

[13] Bawady A, Allam A, Karamany M, Farag H (2023). Tight rope versus screw fixation in ankle syndesmotic injuries. Benha Med J.

[14] Naqvi GA, Cunningham P, Lynch B, Galvin R, Awan N (2012). Fixation of ankle syndesmotic injuries: comparison of tightrope fixation and syndesmotic screw fixation for accuracy of syndesmotic reduction. Am J Sports Med.

[15] Zhang P, Liang Y, He J, Fang Y, Chen P, Wang J (2017). A systematic review of suture-button versus syndesmotic screw in the treatment of distal tibiofibular syndesmosis injury. BMC Musculoskelet Disord.

[16] Degroot H, Al-Omari AA, El Ghazaly SA (2011). Outcomes of suture button repair of the distal tibiofibular syndesmosis. Foot Ankle Int.

[17] Cottom JM, Hyer CF, Philbin TM, Berlet GC (2008). Treatment of syndesmotic disruptions with the Arthrex Tightrope: a report of 25 cases. Foot Ankle Int.

